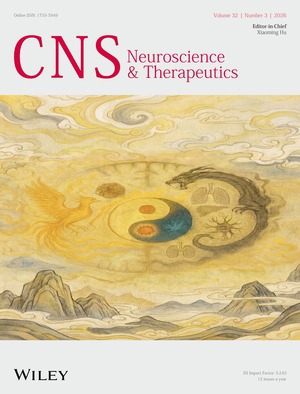# Front Cover

**DOI:** 10.1002/cns.70832

**Published:** 2026-03-10

## Abstract

The cover image is based on the article *Pharmacovigilance Insights Into Immune Checkpoint Inhibitor‐Induced Risk of Paraneoplastic Syndrome: A Large‐Scale Real World Study* by Bufu Tang et al., https://doi.org/10.1002/cns.70747.